# Stereotactic Radiotherapy for Pancreatic Cancer: A Single-Institution Experience

**DOI:** 10.7759/cureus.10618

**Published:** 2020-09-23

**Authors:** Rachel M Glicksman, Hans Chung, Sten Myrehaug, Darby Erler, Renee Korol, Aliaksandr Karotki, Aman Taggar, Yee C Ung

**Affiliations:** 1 Department of Radiation Oncology, University of Toronto, Toronto, CAN; 2 Department of Radiation Oncology, Sunnybrook Health Sciences Centre/University of Toronto, Toronto, CAN; 3 Department of Medical Physics, Sunnybrook Health Sciences Centre/University of Toronto, Toronto, CAN

**Keywords:** pancreatic cancer, sbrt, sabr

## Abstract

Introduction

Despite treatment advances, the prognosis of locally advanced pancreatic cancer is poor. Treatment remains varied and includes systemic and radiotherapy (RT). Stereotactic body radiotherapy (SBRT), highly conformal high-dose RT per fraction, is an emerging treatment option.

Materials and methods

We performed a single-institution retrospective review of patients with pancreatic adenocarcinoma treated with SBRT from 2015-2017. The median dose was 27 Gy (range: 21-36 Gy) in three fractions. Endpoints included local progression (RECIST 1.1; Response Evaluation Criteria in Solid Tumors 1.1), distant metastasis, overall survival, and toxicity.

Results

Forty-one patients were treated, with a median follow-up of eight months. Patients who received SBRT had unresectable (49%), metastatic (17%), or borderline resectable (7%) disease, declined surgery (17%), medically inoperable (7%), or developed local recurrence following the Whipple procedure (2%). The six-month and one-year rates of local progression-free survival, distant metastasis-free survival, and overall survival were 62% and 55%, 44% and 32%, and 70% and 49%, respectively. Five patients (12%) experienced seven late gastrointestinal (GI) grade 3 events.

Conclusion

SBRT may be considered a treatment option to achieve local control of pancreatic cancer and is associated with a modest risk of severe late GI toxicities. Systemic therapies remain important, given the proportion of patients who develop distant metastases.

## Introduction

Pancreatic cancer is the third leading cause of cancer-related death in the United States, with a five-year overall survival of approximately 8% [1-2]. While surgery is the only potentially curative option, many patients are not eligible either because the disease is locally advanced or the patient is medically inoperable. In these cases, the prognosis of clinically localized or locally advanced pancreas cancer (LAPC) remains poor. Treatment for unresectable LAPC remains controversial and includes both systemic therapy and radiotherapy [3]. The LAP07 trial reported an improvement in local control for the chemoradiotherapy (CRT) arm as compared to the chemotherapy alone arm; however, there was no difference in overall survival [4].

Although continuous improvements in systemic therapy is allowing for prolonged survival, local failure remains a clinical problem leading to morbidity and death [5]. Given improvements in overall survival, the need for improved local control is becoming increasingly important. The role of stereotactic body radiotherapy (SBRT) is being investigated as an alternative to long-course chemoradiation in select patients. Advantages of SBRT include improved patient tolerability, shorter treatment time, and, therefore, less time off systemic therapy, as well as safely delivered highly conformal dose-escalation designed to maximize local control [6-15]. The purpose of this study is to report our early institutional real-world outcomes of local control, distant metastasis, overall survival, and toxicity in patients receiving pancreatic SBRT.

## Materials and methods

Patient population

This was a retrospective analysis of consecutive patients treated with pancreatic SBRT at the Odette Cancer Centre, Sunnybrook Health Sciences Centre, Canada, from May 2015 to December 2017. Institutional research ethics board approval was obtained from Sunnybrook Health Sciences Centre (171-2018). Patients were required to have either biopsy-proven pancreatic adenocarcinoma, evidence of a pancreatic mass on imaging associated with significant fludeoxyglucose (FDG) avidity on positron emission tomography-computed tomography (PET-CT) or a pancreatic mass with CT imaging characteristics consistent with adenocarcinoma and a markedly elevated CA-19-9 (>500).

All patients considered for SBRT were evaluated by a hepatobiliary surgeon and deemed unresectable or medically inoperable. All relevant diagnostic scans were centrally reviewed by a radiologist to determine the extent of the disease. Patients with tumors 6 cm or smaller, with Karnofsky Performance status ≥60, were eligible for SBRT. Patients with metastatic disease were eligible for SBRT in settings where local control was deemed important and was reviewed on a case-by-case basis with multi-disciplinary input. The proximity of the tumor to gastrointestinal organs did not preclude treatment with SBRT but did impact the prescription dose, as described below. Patients could not receive chemotherapy within two weeks before or after SBRT. The laboratory values required to proceed with SBRT included neutrophils ≥1,500 cells/mm^3^, platelets ≥80,000 cells/mm^3^, and total bilirubin, aspartate aminotransferase (AST), alanine transaminase (ALT), and alkaline phosphatase each less than three times the institutional limit.

Patients were assessed in follow-up on a three-monthly basis, with history, physical examination, bloodwork, and CT imaging, or sooner as clinically indicated.

SBRT planning details

**Figure 1:**
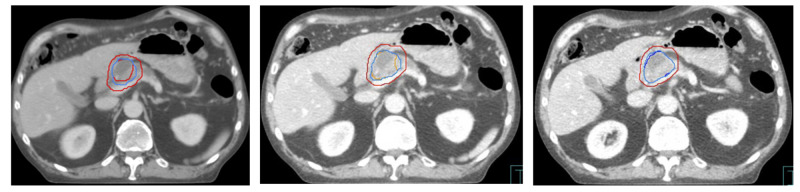
Target definition outline on 4D computed tomography simulation Panel A: Average data set. Inner red: GTV; light blue: ITV; outer red: PTV Panel B: Maximum exhale data set. Orange: GTV; light blue: ITV; outer red: PTV Panel C: Maximum inhale data set. Dark blue: GTV; light blue ITV; outer red: PTV 4D: 4-dimensional; GTV: gross tumour volume; ITV: internal target volume; PTV: planning target volume

Patients were required to have a biliary stent in situ or fiducial markers implanted prior to simulation to facilitate image guidance. Stents were the primary form of surrogate initially, as we did not have institutional access to fiducial markers. The stents were placed directly adjacent to the tumors. A consistent gastric filling protocol was required, and all patients were given oral and intravenous (IV) contrast prior to simulation. Patients were immobilized using an abdominal compression plate to minimize motion due to respiration and a contrast-enhanced four-dimensional computed tomography (4DCT) scan was acquired. Target volumes were contoured on the average, maximal inhale, and maximal exhale 4DCT data sets. The gross tumor volume (GTV) was the macroscopic tumor as defined on imaging and endoscopy. No additional clinical target volume (CTV) was defined. An internal target volume (ITV) was created by combining all GTV contours from each of the datasets. A uniform 0.5 cm expansion around the ITV was added to create the planning target volume (PTV), as per the institutional abdominal SBRT protocol (Figure 1). Mandatory contoured organs at risk (OARs) included the uninvolved pancreas, stomach, duodenum, small bowel, liver, kidneys, spinal canal, heart, and skin. 

**Figure 2:**
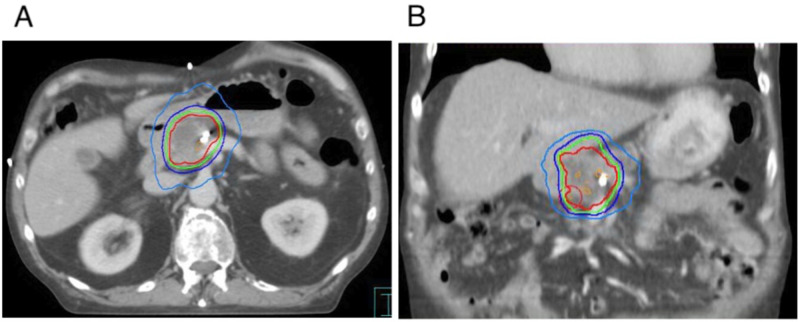
Dose distribution of pancreas stereotactic body radiotherapy plan on axial (A) and coronal (B) computed tomography simulation scan (prescription: 27 Gy in three fractions) Isodose lines: Orange: 27.81 Gy (103% of prescription); red: 27.0 Gy (100% of prescription); green: 25.65 Gy (95% of prescription); dark blue: 21.6 Gy (80% of prescription); light blue: 13.5 Gy (50% of prescription)

Treatment plans were generated in the Pinnacle treatment planning system (Philips Medical Systems, Madison, WI) based on the average 4DCT image sequence, using volumetric modulated arc therapy (VMAT) with one full arc and additional partial arcs if necessary to achieve adequate target coverage and dose constraints (Figure 2). Dose-volume constraints to organs at risk are detailed in Table 1. Dose/fractionation ranged from 21-36 Gy in three fractions, with the aim to treat to as high a dose as possible within this range as determined by dose constraints to OARs (Table 1), with treatment delivered every other day over one week. PTV coverage was V95% ≥99% and V110% <1%. Co-registration of cone-beam CT (CBCT) was performed at each treatment with initial alignment based on a soft tissue match, with fine-tuning based on either fiducial markers or stent as a surrogate [16-17]. SBRT details are outlined in Table 2.

**Table 1:** Dose-volume constraints to organs at risk ITV: internal target volume

Organ	Dose-Volume Constraint
Liver minus ITV	700cc <15Gy
Unilateral kidney	V12Gy <25%
Bilateral kidneys	V15Gy <35%
Spinal canal	V18Gy <0.35cc, V12.3Gy <1.2cc
Stomach	V30Gy <0.5cc, V22.5Gy <5cc
Duodenum	V30Gy <0.5cc, V16.5 <5cc
Small bowel	V30Gy <0.5cc, V24Gy <20cc
Large bowel	V30Gy <0.5cc, V24Gy <20cc
Skin	Dmax <33Gy, V30Gy <10cc

**Table 2:** Stereotactic body radiotherapy details Gy: Gray; BED: biologically effective dose; PTV: planning target volume; CT: computed tomography

Parameter	Result
Radiation dose, n (%) (Gy)	
21	1 (2%)
24	14 (34%)
27	12 (29%)
30	9 (22%)
33	3 (7%)
36	2 (5%)
Fractions	3
Median BED10 (range) (Gy)	51.3 (35.7-79.2)
Median PTV (range) (cc)	78.66 (27.1-183.58)
Median duodenal V30Gy (range) (cc)	0 (0-0.52)
Median duodenal V16.5Gy (range) (cc)	9.08 (0-18.7)
Active breath control, n (%)	
Yes	1 (2%)
No	40 (98%)
Localization to cone beam CT, n (%)	
Stent	25 (61%)
Fiducials	9 (22%)
Surgical clips	3 (7%)
None	3 (7%)
Calcifications	1 (2%)

Outcome criteria

Local response to SBRT was assessed using RECIST (Response Evaluation Criteria in Solid Tumors) version 1.1 criteria [18]. Progressive disease (PD) was defined, as at least a 20% increase in the sum of diameters of target lesions, taking as reference the smallest sum on study, with the sum also demonstrating an absolute increase of at least 5 mm. Toxicity was graded according to the National Cancer Institute Common Terminology Criteria for Adverse Events (NCI CTCAE) version 4.0 scale [19]. Response and toxicity were recorded prospectively in patient records during routine follow-up visits and were collected retrospectively for this study.

Statistical analysis

Kaplan-Meier curves were generated to calculate local progression-free survival, metastasis-free survival, and overall survival, calculated from the start date of SBRT. Patients who were still alive or who had not experienced the event of interest by October 1, 2018, were censored. Statistical routines were performed using SPSS (SPSS Statistics, Version 21.0, Armonk, NY: IBM Corporation).

## Results

Forty-one patients were treated at our center with pancreatic SBRT (Table 3). Median follow-up from the start date of SBRT was eight months (range: 0-21 months) for the entire cohort and 12 months (range: three to 21 months) for patients who are alive.

**Table 3:** Baseline patient characteristics ECOG: Eastern Cooperative Oncology Group; AJCC: American Joint Committee on Cancer; SBRT: stereotactic body radiotherapy

Parameter	Result
Median age (range)	77 (57-90)
Female: male, n (%:%)	23: 19 (55: 45)
ECOG status, n (%)	
0	16 (39%)
1	12 (29%)
2	1 (2%)
3	2 (5%)
4	0 (0%)
Missing	10 (24%)
AJCC 7^th^ edition stage, n (%)	
1A-2B	12 (29%)
3	22 (54%)
4	7 (17%)
Mechanism of diagnosis, n (%)	
Biopsy	27 (66%)
Imaging +/- CA19-9	14 (34%)
Reason for SBRT, n (%)	
Unresectable	20 (49%)
Metastatic disease	7 (17%)
Declined surgery	7 (17%)
Borderline resectable	3 (7%)
Medically inoperable	3 (7%)
Local recurrence following Whipple procedure	1 (2%)
Median tumor size (range) (cm)	2.9 (1.7-5.6)
Location of pancreatic tumor, n (%)	
Head/body	38 (93%)
Tail	3 (7%)
Chemotherapy, n (%)	
Prior to SBRT	12 (29%)
Concurrent	0 (0%)
Following SBRT	10 (24%)
Median follow-up (range) (months)	8 (0-21)

Local control and survival outcomes

At the time of the last follow-up, 13 patients (31.7%) developed local progression, at a median time of three months (range: 1-12 months) following SBRT. Of these 13 patients, nine developed metastases (at the same time as local progression in eight of these patients and subsequent to local progression in one patient), whereas two had metastatic disease at initial presentation. In total, 23 patients (56%) had metastatic disease at last follow-up, including seven patients who had synchronous metastases at diagnosis. Twenty-six patients (63%) had died at the time of the last follow-up.

**Figure 3:**
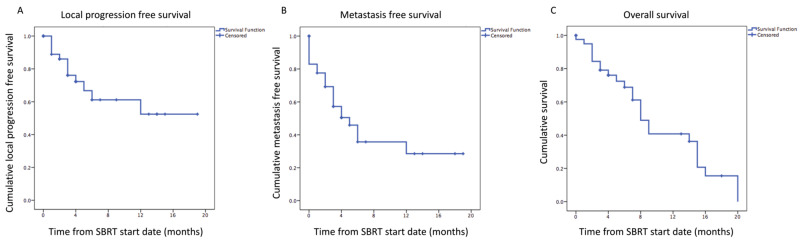
Kaplan-Meier univariate analyses of local progression-free survival (A), metastasis-free survival (B) and overall survival (C)

The six-month and one-year local progression-free survival rate respectively was 62% and 55% (Figure 3A), the metastasis-free survival rate was 44% and 32% (Figure 3B), and overall survival was 70% and 49% (Figure 3C).

Toxicities

Acute toxicities occurred in 14 patients (34.1%) of which nine experienced grade one abdominal pain, four experienced grade one fatigue, and one both. 

Late toxicities were observed in five patients (12.2%) with a total of seven grade three gastrointestinal (GI) events, which may be treatment-related. These events include duodenal stenosis (two events), a fistula between the cancer and the duodenum (two events), portal vein stenosis (one event), gastric outlet obstruction (one event), and duodenal ulcer resulting in hemorrhage (one event). No patients experienced grade four-five late GI toxicity. Given the difficulty in ascertaining whether an event in the location of the pancreas is due to tumor progression or treatment-related toxicity, if we exclude patients with local progression from measures of late toxicity, two patients (4.9%) experienced a total of three events, including duodenal ulcer resulting in hemorrhage (one event), mass-duodenal fistula (one event), and gastric outlet obstruction (one event). 

Chemotherapy details

Prior to SBRT, 12 patients (29.3%) received chemotherapy; nine patients received FOLFIRINOX (folonic acid, fluorouracil, irinotecan, and oxaliplatin), one received gemcitabine-nab-paclitaxel, and two received gemcitabine alone. Following SBRT, 10 patients (24.4%) received chemotherapy, of which three continued on FOLFIRINOX, two received gemcitabine-Abraxane, and five received gemcitabine alone.

Surgical details

One patient underwent a Whipple procedure 31 months prior to SBRT, with pathology significant for an approximate 4 cm margin-negative grade two pancreatic adenocarcinoma pT3N0, with 12 lymph nodes dissected. This patient also completed six months of adjuvant gemcitabine. The patient subsequently developed locally recurrent disease and underwent SBRT. In addition, two patients who underwent SBRT for borderline resectable disease were able to proceed with the Whipple procedure. The first patient had surgery performed one and a half months following the completion of SBRT, with pathology revealing a 2.8 cm margin-negative grade two ductal adenocarcinoma, pT3N0, with 22 lymph nodes removed. The second patient underwent both the Whipple procedure and NanoKnife surgery, with pathologic assessment of the specimen revealing no evidence of residual disease.

## Discussion

The treatment of unresectable LAPC remains controversial. SBRT is increasingly used in these patients; however, its exact role and the optimal sequencing in the context of other treatments are still unknown. Until the results of the currently ongoing phase III Pancreatic Cancer Radiotherapy Study Group Trial evaluating patients with LAPC treated with modified FOLFIRNOX with or without SBRT (ClinicalTrials.gov NCT01926197) are published, treatment will be based on clinical experience derived from single institutional series. In this report, we highlight the outcomes of real-world patients treated with SBRT at our institution, which can add to the available body of evidence.

Local control reported in this study was 62% at six months and 55% at one year. Compared to other series, this is a relatively low rate of local control. In a systematic review of 13 studies and 889 patients treated with pancreatic SBRT for patients with locally advanced disease, the one-year local control rate was 72% [15]. This review found that the total dose delivered and a higher number of fractions were significantly associated with one-year locoregional control [15]. Our study employed a relatively low total dose (median 27 Gy) with a moderate number of fractions (three fractions), for a median BED10 of 51.3 Gy. Comparatively, studies with higher reported local control rates utilized a higher total dose and fractionation for a higher calculated BED10. For example, Chuong et al. treated 73 patients with LAPC or borderline resectable pancreas cancer with a median dose of 35 Gy in five fractions (median BED10 of 59.5 Gy) with a local control rate in nonsurgical patients of 81% at one year [20]. Similarly, Song et al. assessed 59 LAPC patients treated with a median dose of 45 Gy in five fractions (median BED10 of 85.5 Gy), reporting freedom from local progression of 90.8% at one year [21].

The role of dose-escalation in LAPC has been studied in the context of fractionated intensity-modulated RT (IMRT) treatment [22]. Of 200 patients with LAPC treated with induction chemotherapy followed by chemoradiation (50.4 Gy in 28 fractions; BED10 of 59.5 Gy), a subset of 47 patients was eligible for dose-escalation with BED10 >70 Gy [22]. Patients who received dose escalation had significantly higher overall survival (17.8 versus 15 months) and improved local-regional recurrence-free survival (10.2 versus 6.2 months) [22].

We initially employed a three fraction treatment regimen based on the available evidence at the time we began the pancreas SBRT program, and to minimize treatment visits for patients, given the palliative nature of treatment [8,23]. However, based on our local control results, and the results of other series above, our revised institutional policy is to escalate the total radiation dose as allowable based on dose-volume constraints to organs at risk and to consider a five fraction treatment regimen (to a dose as high as allowable between 30 and 50 Gy; BED10 of 48-100 Gy) as a means to try to improve local control and reduce late GI toxicity.

Late grade three GI toxicity was observed in 12% of patients, which is similar to two other published studies [24-25]. One previous analysis has shown that the radiotherapy dose to the duodenum was significantly related to toxicity [26]. In our study, we used two dose-volume constraints each for the duodenum and stomach (Table 1). In one patient (2%), we exceeded the institutional duodenal V30Gy <0.5 cc dose constraint and the patient received V30Gy 0.52 cc. However, in the remainder of patients, we were able to meet this dose constraint by reducing the total prescription dose. There was more difficulty in achieving the V16.5Gy <5 cc dose constraint, as recommended by the American Association of Physicists in Medicine (AAPM) Task Group 101 and consensus guidelines, with a median V16.5Gy of 9.08 cc [27-28]. This constraint was exceeded in 34 patients (83%) based on the very close proximity of the pancreatic lesion to the duodenum. All cases were presented and reviewed at quality assurance rounds prior to treatment delivery whereby a discussion regarding the risks of decreasing the total prescription dose to achieve this dose constraint versus the risk of decreasing local control with a lower total prescription dose occurred. Ultimately, further work regarding optimal dose-volume constraint metrics and their relationship with late toxicity outcomes is necessary. In addition, further follow-up in our study cohort is needed to ascertain the true rates of late toxicity.

This study has some limitations. First, the patient sample is small, and there is significant heterogeneity in the patients included in terms of diagnostic stage, reasons for receiving SBRT, the dose of SBRT received, and the use of chemotherapy. However, the heterogeneity is reflective of patients in real-world settings who received SBRT at a large, tertiary cancer center, and the study included all patients treated with SBRT at our institution from May 2015 to December 2017. In addition, the heterogeneity is reflective of current guidelines with no clear consensus on treatment for this group of patients [3]. Further research is needed to guide patient selection for various treatments in this population. Second, the median follow-up time is short and does not allow for an analysis of fully mature results, including fully mature late toxicity results. Third, the use of the RECIST v1.1 definition for local progression is not a perfect endpoint. Throughout other published studies reporting on pancreas SBRT, there are a variety of endpoints used for local progression assessment, with a lack of consensus. In particular, the RECIST definition can be problematic given the potential increase in tumor size in the approximately three-month period following pancreas SBRT secondary to tumoral edema. This may have led to an incorrect classification of local progression in patients who did not truly experience local progression (i.e. false positive).

## Conclusions

Our results suggest that SBRT may be considered a treatment option in patients where local control of their pancreas tumor is important as part of their comprehensive multi-modality management. Based on our results, the use of a multi-fraction regimen and high total dose as allowable based on dose-volume constraints to organs at risk should be considered to minimize toxicity and improve local control outcomes. Further research is needed to improve outcomes for these patients.
